# Increased serum AXL is associated with radiographic knee osteoarthritis severity

**DOI:** 10.1111/1756-185X.14239

**Published:** 2021-11-28

**Authors:** Shao Zhenghai

**Affiliations:** ^1^ Shanghai Kaiyuan Orthopedic Hospital Shanghai China

**Keywords:** AXL, correlation, osteoarthritis, serum

## Abstract

**Objective:**

To investigate the expression and clinical significance of serum soluble AXL in patients with radiographic knee osteoarthritis (KOA).

**Methods:**

There were 183 patients with KOA who were selected and divided based on the Kellgren‐Lawrence (KL) score into KL 0 subgroups (n = 42), KL I‐II subgroups (n = 90), and KL III‐IV subgroups (n = 51). Healthy volunteers (n = 170) in our hospital were selected with matched age and gender as the control group. AXL level in serum was detected by enzyme‐linked immunosorbent assay. The correlation between serum AXL with severity and clinical indicators of osteoarthritis was analyzed.

**Results:**

The level of serum AXL was significantly higher in the osteoarthritis group than that in the control group (*P* < .05). In the osteoarthritis patients, serum AXL level was increased with the increase of KL score. Serum AXL level was positively correlated with age, body mass index, erythrocyte sedimentation rate, serum C‐reactive protein, cartilage oligomeric protein, matrix metalloproteinase‐13, and transforming growth factor‐β1 levels. The cut‐off value for serum AXL was determined as 33.375 ng/mL by receiver operating curve analysis.

**Conclusion:**

The level of serum AXL in patients with osteoarthritis is significantly higher than in healthy controls, and is closely related to the severity of radiographic osteoarthritis.

## 1 INTRODUCTION

Knee osteoarthritis (KOA) has become the fastest basis of expanding physical disability with a significant economic burden worldwide.^1^, ^2^, ^3^ KOA is the most common form of arthritis and is characterized by joint degeneration, loss of cartilage, osteophyte formation, cysts, and alterations of subchondral bone.^4^, ^5^ Further, a broad range of mechanical and biochemical inflammatory mediators (pro‐inflammatory cytokines, growth factors, and matrix metalloproteinase) contribute to the pathogenesis of KOA.^6^, ^7^ To date, the prime cause of KOA development remains unidentified and optimal treatment remains elusive. Studies show that more than 40 million Americans having OA, and 80% of them are older than 50 years.^8^ Other studies show that KOA prevalence rate in the Chinese and Japanese populations is also cumulative. The prevalence rate in the Chinese population reached 15.6% in those over 40 years.^9^ The prevalence rate of KOA in the Japanese population was reported as up to 42.0% in men and 62.4% in women aged over 40 years.^10^ Therefore, KOA is a global issue in the elderly population and early diagnosis is required to begin possible treatment.

The radiography imaging technique is viewed as a gold standard method for diagnosis of KOA, but the current imaging technique is suffering from sensitivity and specificity.^11^ Imaging technique allows detection of OA of the knee joint space narrowing, presence of osteophytes, subchondral sclerosis, and cysts. Due to the lack of sensitivity and specificity of radiographic imaging techniques, there is an urgency to develop a potential alternative tool for the diagnosis of KOA. Body fluid serum is routinely tested in clinics for diagnosis, and treatment of different diseases. This is a very decisive medium and harbors plenty of biomarkers for the monitoring of our health. Several biomarkers are known to be correlated with the extent of OA on radiography of the knee and are being proposed as diagnostic tools.^12^, ^13^, ^14^, ^15^ However, the currently used biomarkers are inadequate for the prognosis of radiographic KOA.

The receptor tyrosine kinase AXL is a 140 kDa protein that belongs to a tyrosine kinase receptor (TAM) subfamily, together with Tyro3 and Mer. The TAM receptors (AXL, Tyro3, and Mer) play a critical role in innate immune homeostasis and vitamin K‐dependent ligand growth arrest‐specific protein 6 (GAS6) can bind all 3 receptors with the highest affinity for AXL.^16^, ^17^ Transmembrane protein AXL can be cleaved proteolytically at its extracellular membrane domain and subsequently released as soluble AXL, which can be detected in serum or plasma.^18^, ^19^ Furthermore, studies revealed that targeted delivery of TAM receptor ligand genes Gas6 diminishes the arthritis pathology effectively but the endogenous role of AXL in arthritis development is not fully understood.^20^ Thus, the present study hypothesized that endogenous AXL concentration may be correlated with the severity of KOA and can predict the development and progression of KOA as seen on radiography of the knee.

In the present study, AXL levels were analyzed in sera from participants suffering from KOA and divided into different groups according to the Kellgren‐Lawrence (KL) score. The diagnostic performance of AXL for KOA in comparison to participant control groups was assessed. Furthermore, the accuracy of AXL in KOA of different groups that demonstrated the potential diagnostic value of AXL for routine clinical use in surveillance of patients at high risk for KOA severity was determined.

## 2 MATERIALS AND METHODS

### 2.1 Subjects

This study included 183 patients with KOA who were admitted to our hospital from July 2019 to December 2020. There were 102 males and 81 females, with a median age of 68 years. Patient inclusion criteria were as follows: (a) all patients were diagnosed with KOA with duration of disease >6 months; (b) all patients had radiological evidence of osteoarthritis with a KL score of 0‐IV. All patients were graded as KL according to the X‐ray pictures of bone and joint: grade 0, no change; grade I, slight osteophyte; grade II, obvious osteophytes, no joint space involved; grade III, moderate narrowing of joint space; grade IV, joint space narrowing, subchondral osteosclerosis.^21^ There were 42 cases of grade 0, 51 cases of grade I, 39 cases of grade II, 28 cases of grade III and 23 cases of grade IV in 183 patients. Patient exclusion criteria are as follows: (a) patients had concurrent systemic or local inflammation, infection, trauma, tumors, connective tissue disease, and autoimmune disease; (b) patients had received any intra‐articular injections within a month or systemic glucocorticoids within 3 months; (c) patients had history of knee injury and operation, rheumatoid arthritis, ankylosing spondylitis, or severe osteoporosis. The serum samples of 170 age and gender matched healthy people in our hospital during the same period were selected as the control group, including 91 males and 79 females, with a median age of 64 years. The age, gender, height, and weight of all participates were collected. This study was approved by Institutional Review Board of the hospital ethics committee. All subjects provided written informed consent before sample collection. All experimental protocols were approved by the ethics committee of the hospital.

### 2.2 X‐ray measurement of knee joint

All subjects were in the weight‐bearing standing position, and the X‐ray films of both knees in the anterior‐posterior position and lateral position were taken respectively. The evaluation of KL grade and double‐blind measurement of joint structure were carried out by 2 experienced rheumatologists. When the evaluation of the 2 specialists was different, the joint KL grade and joint structure were evaluated by senior specialists in the Department of Radiology.

### 2.3 Specimen collection and preparation

Whole blood was collected from fasting participants in the morning. Blood samples were centrifuged (15 000 × g for 10 minutes at 4°C) to separate serum. The processed serum supernatant was then aliquoted into a 1.5 mL Eppendorf tube and stored at −80°C for further analysis.

### 2.4 Measurement of serum proteins and cytokines concentration

The serum concentrations of cytokines and proteins were determined using enzyme‐linked immunosorbent assay (ELISA) kits, including serum C‐reactive protein (CRP) (DCRP00, R&D Systems), cartilage oligomeric protein (COMP) (DCMP0), matrix metalloproteinase‐13 (MMP‐13) (DY511), transforming growth factor‐β1 (TGF‐β1) (DY240), Gas6 (DY885B), and AXL (DAXL00), according to the manufacturer's protocol. In brief, serum samples were directly transferred or diluted to the wells of the ELISA plate, and the absorbance was measured in a microplate reader. The protein levels were quantified by their corresponding standard curve.

### 2.5 Statistical analysis

SPSS 20.0 software was used for statistical analysis. The measurement data were expressed as median (interquartile range), and analyzed by one‐way analysis of variance or Mann‐Whitney *U* test. The correlation between serum AXL level and other clinical indicators was analyzed by Spearman correlation. Receiver operating curve (ROC) analysis was carried out to determine the diagnostic potential of AXL for KOA; the difference was statistically significant at *P* < .05.

## 3 RESULTS

### 3.1 Baseline demographic and clinical characteristics of subjects

A total of 183 OA patients were enrolled in this study. The baseline demographic and clinical characteristics are shown in Table 1. There was no significant difference in age and gender between the control group and OA group. The mean body mass index (BMI) and erythrocyte sedimentation rate (ESR) were significantly higher in OA patients with respect to healthy controls (both *P* < .05). Analysis of biochemical parameters revealed significantly higher serum CRP, COMP, MMP‐13, TGF‐β1 and Gas6 levels in the OA group compared to control group (all *P* < .05).

**TABLE 1:** Demographic and clinical characteristics of osteoarthritis patients and healthy controls

Clinical parameters	Control (n = 170)	Osteoarthritis (n = 183)	*P* value
Age	66.0 (59.8‐72.0)	68.0 (61.0‐74.0)	.223
Gender, male	91 (53.5%)	102 (55.7%)	.677
BMI, kg/m^2^	23.8 (21.4‐25.5)	25.0 (23.4‐26.4)	<.001
ESR, mm/h	8.0 (6.8‐9.3)	18.1 (16.9‐20.2)	<.001
CRP, μg/mL	3.4 (3.2‐3.6) CV: 0.120	6.0 (5.6‐6.6) CV: 0.113	<.001
COMP, ng/mL	14.4 (13.3‐15.6) CV: 0.105	25.8 (23.8‐28.7) CV: 0.141	<.001
MMP‐13, ng/mL	12.5 (11.5‐13.9) CV: 0.143	21.8 (19.0‐23.7) CV: 0.152	<.001
TGF‐β1, pg/mL	12.5 (11.1‐13.8) CV: 0.148	16.6 (15.2‐18.8) CV: 0.161	<.001
Gas6, ng/mL	16.6 (15.0‐18.3) CV: 0.151	24.6 (22.1‐26.9) CV: 0.135	<.001

Abbreviations: BMI, body mass index; COMP, cartilage oligomeric protein; CRP, C‐reactive protein; ESR, erythrocyte sedimentation rate; Gas6, growth arrest‐specific gene 6 protein; CV, coefficient of variation; MMP‐13, matrix metalloproteinase‐13; TGF‐β1, transforming growth factor‐β1. Mann‐Whitney test or χ^2^ test was performed

### 3.2 Serum AXL concentrations were elevated in KOA patients

**FIGURE 1:**
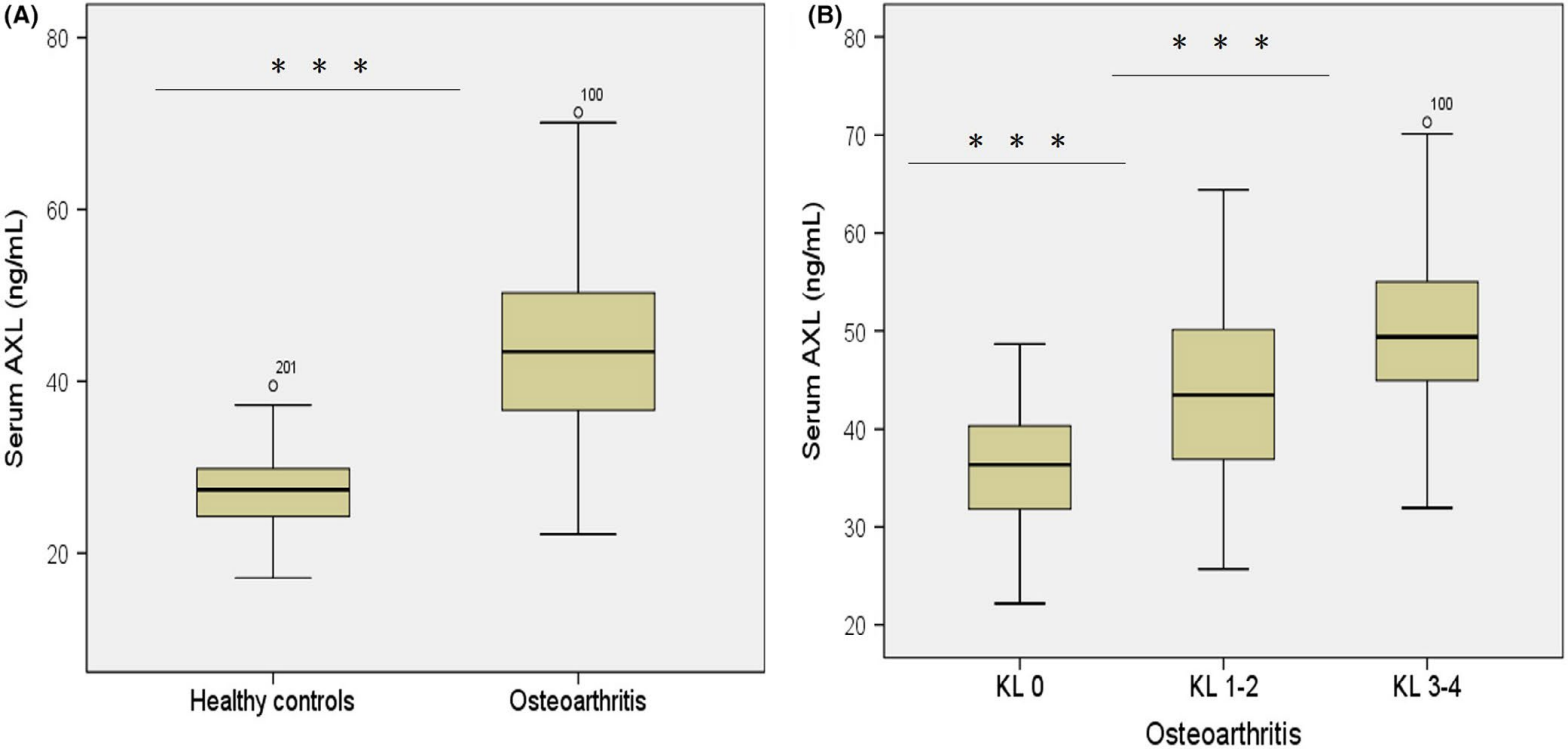
Serum AXL levels in osteoarthritis (OA) patients. (A) Comparison of serum AXL between patients with knee OA (Kellgren‐Lawrence [KL] grades I‐III) and healthy subjects. (B) Comparison of serum AXL between KL 0, KL I‐II, and KL III‐IV in patients with knee OA. ***P < .001

The serum AXL concentrations were analyzed in KOA patients and healthy controls by non‐parametric tests. Serum AXL levels were analyzed between males and females. The serum AXL level was significantly higher in the OA patients (43.45 [36.61‐50.33] ng/mL, coefficient of variation [CV]: 0.213) compared with that in the healthy control (27.38 [24.25‐29.82] pg/mL, CV: 0.155) (*P* < .001; Figure 1A). Also, serum AXL levels were significantly higher in KOA compared to healthy controls, after BMI adjustments (Table S1). Moreover, among OA patients, serum AXL levels were significantly higher in subjects with KL I‐II grade compared to subjects with KL 0 grade, higher in subjects with KL III‐IV compared to subjects with KL I‐II grade (both *P* < .001; Figure 1B). Additionally, significant difference was found in serum AXL and different KL grades after BMI adjustment (Table S2). A Mann‐Whitney test was performed to compare the serum AXL levels between males and females. There was no significant difference observed between male and female groups (Table S3).

### 3.3 Correlation between serum AXL level and clinical indicators

**FIGURE 2:**
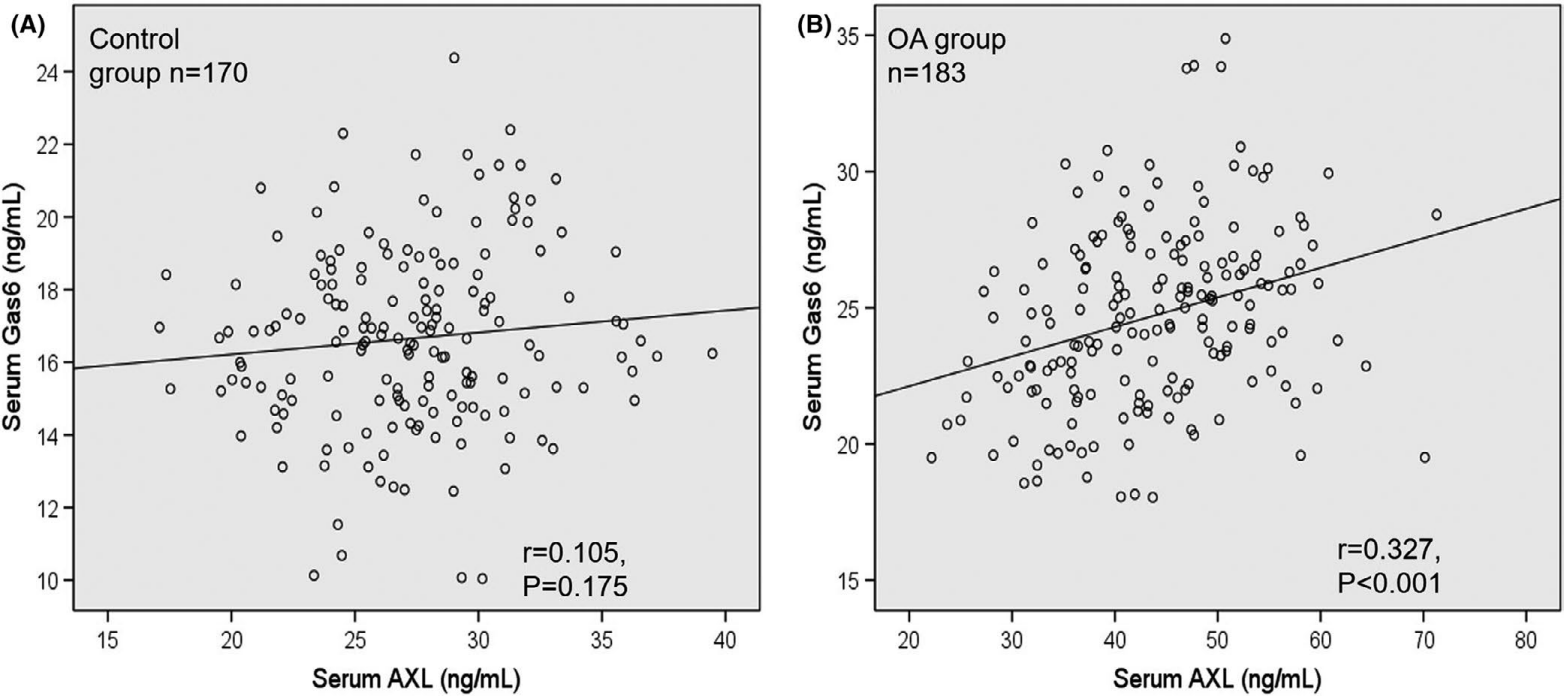
The correlation between serum AXL and clinical parameters in osteoarthritis (OA) patients. Spearman correlation test was performed. ***P < .001

Spearman correlation test was used to determine the relationship between serum AXL and other biochemical indexes. In OA patients, serum AXL was significantly positively correlated with BMI, ESR, CRP, COMP, MMP‐13 and TGF‐β (Table 2). There was no significant correlation between serum AXL and Gas6 in the control group (r = 0.105, *P* =.175; Figure 2A). However, in the OA group, serum AXL showed significant positive correlation with Gas6 (r = 0.327, *P* < .001) (Figure 2B).

**TABLE 2:** Correlation between serum AXL and clinical indicators

	r	*P*
Age	0.058	.437
BMI (kg/m^2^)	0.149	.044
ESR (mm/h)	0.210	.004
CRP (μg/mL)	0.221	.003
COMP (ng/mL)	0.190	.010
MMP‐13 (ng/mL)	0.287	<.001
TGF‐β1 (pg/mL)	0.192	.009

Note

Spearman correlation was performed.

Abbreviations: BMI, body mass index; COMP, cartilage oligomeric protein; CRP, C‐reactive protein; ESR, erythrocyte sedimentation rate; MMP‐13, matrix metalloproteinase‐13; TGF‐β1, transforming growth factor‐β1.

### 3.4 Diagnostic potential of AXL for radiographic KOA

**FIGURE 3:**
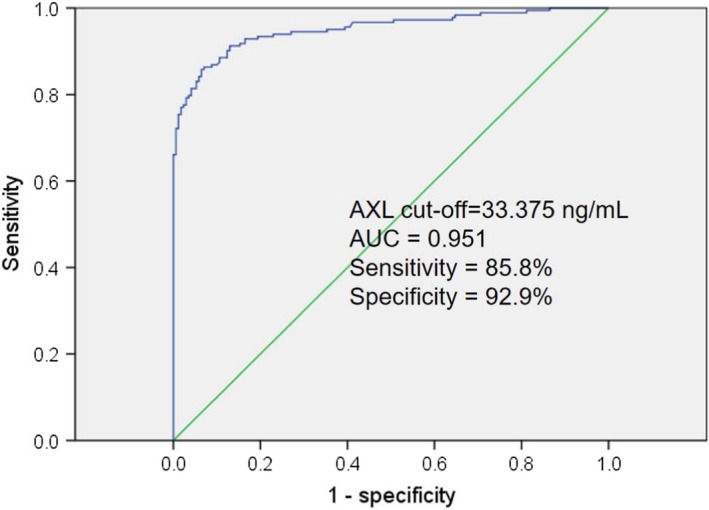
Receiver operating curve analysis of serum AXL levels discriminating between patients with knee osteoarthritis (OA) and healthy subjects

Then the ROC curve was drawn. The optimal cut‐off value of serum AXL level in the diagnosis of osteoarthritis was 33.375 ng/mL, with 85.8% sensitivity and 92.9% specificity (area under the curve [AUC] = 0. 951, 95% CI = 0.929‐0.973; *P* < .001) (Figure 3).

## 4 DISCUSSION

Clinical findings are more important than radiographs in KOA to decide further course of management.^22^, ^23^, ^24^ Radiographs alone do not reflect the clinical severity of KOA and routine use of radiographs in the management of KOA is not recommended.^23^ In this study, the clinical value of AXL serum levels was used with other biochemical molecules (CRP, COMP, MMP‐13, TGF‐β, and Gas6) for diagnosis and assessment of disease severity of KAO.

The baseline analysis and biochemical parameters revealed that OA patients have a significantly higher concentration of CRP, COMP, MMP‐13, TGF‐β1, and Gas6 compared to the control group (Table 1). These findings are in line with several other studies that indicate that these biochemical parameters are involved in osteoarthritis development and treatments.^25^, ^26^, ^27^, ^28^, ^29^ In addition, serum AXL level in OA patients was significantly higher compared with that in the healthy controls (Figure 1A). Thus, correlation between serum AXL level and biochemical indicators can distinguish KOA patients from healthy controls. Furthermore, Spearman correlation analysis revealed that serum AXL level is positively correlated with the clinical biochemical indicators BMI, ESR, CRP, COMP, MMP‐13, and TGF‐β (Table 2). Moreover, a significant positive correlation was observed between serum level AXL and Gas6 in OA patient groups and no correlation among control groups (Figure 2). Besides that, serum AXL showed a diagnostic accuracy with an AUC of 0.951 (cut‐off 33.37 ng/mL) for the detection of osteoarthritis with 85.8% sensitivity and 92.9% specificity (AUC= 0. 951, 95% CI = 0.929‐0.973; *P* < .001) compared to healthy controls.

The current study revealed positive correlations among serum AXL levels and the radiographic severity of KOA, and KL score. AXL expression increased with increasing radiographic disease severity (KL grade). The AXL level was significantly higher in subjects with KL I‐II grade compared to subjects with KL 0 grade, higher in subjects with KL III‐IV compared to subjects with KL I‐II grade. The optimal cut‐off value of serum AXL level was obtained by ROC curve as 33.375 ng/mL, which was used to distinguish healthy controls and all osteoarthritis (including KL 0 to IV) (Figure 3). Thus, based on our biochemical analysis, we revealed that the AXL cut‐off value could be useful during screening for radiographic KOA. Serum AXL level might be an indicator of radiographic KOA diagnosis and severity assessments.

## 5 CONCLUSION

Serum AXL levels were significantly elevated in KOA patients and have a positive correlation between serum AXL levels with the degree of severity in KOA patients. Measurement of AXL level in the serum can be used as an alternative biomarker to assess the progression of OA in addition to the use of traditional methods for assessing the risk and severity of KOA. The diagnostic accuracy of AXL in radiographic KOA should be confirmed in different patient cohorts.

## CONFLICT OF INTEREST

The authors declare no competing financial interests.

## ETHICS APPROVAL

Our study has been approved by the medical ethics committee of the Shanghai Kaiyuan Orthopedic Hospital and written informed consents were obtained from all included patients.

## Supporting information

Tab S1‐S3

## Data Availability

The datasets used and/or analyzed during the current study are available from the corresponding author on reasonable request.
