# Supplementary material for: Increased serum AXL is associated with radiographic knee osteoarthritis severity
Source: Int J Rheum Dis. 2021 Nov 28;25(1):32–7. doi: 10.1111/1756-185X.14239 (PMC9298778; doi:10.1111/1756-185X.14239)
Supplement: Supplementary file 1 — Tab S1‐S3 [file APL-25-32-s001.docx]

**Supplementary information for**

**Increased serum AXL is associated with radiographic knee osteoarthritis severity**

Zhenghai Shao ^a,*^

Shanghai Kaiyuan orthopedic hospital, Shanghai, 200135, China

* Corresponding Authors: Zhenghai Shao, Director, Department of orthopedics, Shanghai Kaiyuan orthopedic hospital, Shanghai, 200135, China. Tel: +8615021006146; Email addresses: 52881725@qq.com

Zhenghai Shao contributed as first author and corresponding author to this work.

**Supplementary Table 1：**Serum AXL is significantly higher in KOA compared to healthy controls after BMI adjustment.

| **Tests of Between-Subjects Effects** | | | | | |
| --- | --- | --- | --- | --- | --- |
| Dependent Variable: AXL | | | | | |
| Source | Type III Sum of Squares | df | Mean Square | F | Sig. |
| Corrected Model | 28401.877^a^ | 2 | 14200.938 | 287.930 | 0.000 |
| Intercept | 3202.447 | 1 | 3202.447 | 64.931 | 0.000 |
| BMI | 76.475 | 1 | 76.475 | 1.551 | 0.214 |
| Grouping | 26915.593 | 1 | 26915.593 | 545.725 | 0.000 |
| Error | 17262.290 | 350 | 49.321 |  |  |
| Total | 511117.338 | 353 |  |  |  |
| Corrected Total | 45664.167 | 352 |  |  |  |
| a. R Squared = 0.622 (Adjusted R Squared = 0.620) | | | | | |
| **Supplementary Table 2:** Significant difference in serum AXL between different KL grades after BMI adjustment.   \| **Tests of Between-Subjects Effects** \| \| \| \| \| \| \| --- \| --- \| --- \| --- \| --- \| --- \| \| Dependent Variable: AXL \| \| \| \| \| \| \|  \| \| \| \| \| \| \| Source \| Type III Sum of Squares \| df \| Mean Square \| F \| Sig. \| \| Corrected Model \| 4639.993^a^ \| 3 \| 1546.664 \| 24.937 \| 0.000 \| \| Intercept \| 2365.241 \| 1 \| 2365.241 \| 38.134 \| 0.000 \| \| BMI \| 28.104 \| 1 \| 28.104 \| 0.453 \| 0.502 \| \| KLClass. \| 4210.018 \| 2 \| 2105.009 \| 33.939 \| 0.000 \| \| Error \| 11102.281 \| 179 \| 62.024 \|  \|  \| \| Total \| 364864.284 \| 183 \|  \|  \|  \| \| Corrected Total \| 15742.274 \| 182 \|  \|  \|  \| \| a. R Squared = 0.295 (Adjusted R Squared = 0.283) \| \| \| \| \| \| | | | | | |

**Supplementary Table 3:** Compare the serum AXL levels between males and females.

**Mann-Whitney Test**

**Ranks**

| Gender | N | Mean Rank | Sum of Rank |
| --- | --- | --- | --- |
| AXL 0  1  Total | 82  101  183 | 95.29  89.33 | 7813.50  9022.50 |

**Test Statistics^a^**

|  | AXL |
| --- | --- |
| Mann-Whitney U  Wilcoxon W  Z  Asymp. Sig. (2-tailed) | 3871.500  9022.500  -0.756  0.449 |

a. Grouping variable: Gender
